# Preconditioning of bone marrow-derived mesenchymal stromal cells by tetramethylpyrazine enhances cell migration and improves functional recovery after focal cerebral ischemia in rats

**DOI:** 10.1186/s13287-017-0565-7

**Published:** 2017-05-12

**Authors:** Lin Li, Lisheng Chu, Yan Fang, Yan Yang, Tiebing Qu, Jianping Zhang, Yuanjun Yin, Jingjing Gu

**Affiliations:** 1 Department of Physiology, Zhejiang Chinese Medical University, Hangzhou, 310053 China; 2 Department of Anatomy, Zhejiang Chinese Medical University, Hangzhou, 310053 China; 3 Department of Pathology, Zhejiang Chinese Medical University, Hangzhou, 310053 China

**Keywords:** Bone marrow-derived mesenchymal stem cells, Tetramethylpyrazine, Preconditioning, Migration, CXCR4

## Abstract

**Background:**

Transplantation of bone marrow-derived mesenchymal stem cells (BMSCs) is one of the new therapeutic strategies for treating ischemic stroke. However, the relatively poor migratory capacity of BMSCs toward infarcted regions limited the therapeutic potential of this approach. Pharmacological preconditioning can increase the expression of CXC chemokine receptor 4 (CXCR4) in BMSCs and enhance cell migration toward the injury site. In the present study, we investigated whether tetramethylpyrazine (TMP) preconditioning could enhance BMSCs migration to the ischemic brain and improve functional recovery through upregulating CXCR4 expression.

**Methods:**

BMSCs were identified by flow cytometry analysis. BMSCs migration was evaluated in vitro by transwell migration assay, and CXCR4 expression was measured by quantitative reverse transcription-polymerase chain reaction and western blot analysis. In rats with focal cerebral ischemia, the neurological function was evaluated by the modified neurological severity score, the adhesive removal test and the corner test. The homing BMSCs and angiogenesis were detected by immunofluorescence, and expression of stromal cell-derived factor-1 (SDF-1) and CXCR4 was measured by western blot analysis.

**Results:**

Flow cytometry analysis demonstrated that BMSCs expressed CD29 and CD90, but not CD34 and CD45. TMP pretreatment dose-dependently induced BMSCs migration and CXCR4 expression in vitro, which was significantly inhibited by AMD3100, a CXCR4 antagonist. In rat stroke models, we found more TMP-preconditioned BMSCs homing toward the infarcted regions than nonpreconditioned cells, leading to improved neurological performance and enhanced angiogenesis. Moreover, TMP-preconditioned BMSCs significantly upregulated the protein expression of SDF-1 and CXCR4 in the ischemic boundary regions. These beneficial effects of TMP preconditioning were blocked by AMD3100.

**Conclusion:**

TMP preconditioning enhances the migration and homing ability of BMSCs, increases CXCR4 expression, promotes angiogenesis, and improves neurological performance. Therefore, TMP preconditioning may be an effective strategy to improve the therapeutic potency of BMSCs for ischemic stroke due to enhanced BMSCs migration to ischemic regions.

## Background

Stroke is a major cause of death and adult disability worldwide. Currently, administration of recombinant tissue plasminogen activator is the sole effective treatment for acute ischemic stroke. However, only approximately 5% of the patients could benefit from this treatment due to the narrow therapeutic time window and the risk of subsequent hemorrhage [1]. Thus, developing restorative therapeutics, particularly stem cell therapy, has attracted great interest [2, 3]. Bone marrow-derived mesenchymal stem cells (BMSCs) represent an ideal candidate for cell transplantation therapy because they are easily obtained and can be expanded rapidly ex vivo [4]. BMSCs transplantation is reported to be safe and effective for the treatment of ischemic stroke [5–7]. However, the low migration efficiency of the transplanted BMSCs toward the injured area limits the effect of BMSC delivery via the vascular route [8, 9]. Thus, strategies promoting BMSCs migration into the peri-ischemic brain tissue may enhance the effectiveness of BMSCs-based therapy [10].

Chemokines play a pivotal role in controlling cellular migration. It has been proven that stromal cell-derived factor-1 (SDF-1) contributes largely to recruitment of stem cells to ischemic tissue by combining with its receptor CXC chemokine receptor 4 (CXCR4) [11, 12]. SDF-1 is upregulated in the ischemic penumbral region after stroke, and the interaction between SDF-1 and CXCR4 triggers BMSCs migration toward the injured area [13–16]. While CXCR4 is highly expressed in BMSCs within the bone marrow, its expression is severely reduced during ex vivo expansion of BMSCs [17, 18]. Such low levels of CXCR4 could decrease the ability of transplanted BMSCs to respond to homing signals emanated from the ischemic tissue. Most MSCs have high intracellular CXCR4 expression, yet only a small percentage of MSCs express CXCR4 on the cell surface [19]. Therefore, how to mobilize the internalized receptor and increase membranous CXCR4 expression becomes critical to improving the engraftment of BMSCs and the benefit of their transplantation. Some studies have shown that cell migration could be improved by preconditioning the stem cells with chemical compounds, cytokines, and hypoxic conditions during the stage of in vitro expansion before transplantation [10].

Tetramethylpyrazine (TMP), a pharmacologically active component extracted from the rhizome of the Chinese herb *Rhizoma Chuanxiong* (Chuanxiong), has been widely used in clinical treatment of cerebrovascular and cardiovascular diseases in China [20–22]. However, the molecular mechanisms involved in the therapeutic effects of TMP are largely unclear. Recently, TMP was found to act as a highly efficient regulator for the migration of various cell types, such as neural precursor cells [23], bone marrow mononuclear cells [24], and brain endothelial cells [25]. These finding suggest that TMP may have the potential to promote the migration of BMSCs. In the present study, we studied the effect of TMP preconditioning on BMSCs migration and homing followed by exploration of the relevant mechanisms, and evaluated the effect of TMP-preconditioned BMSCs on the neurological function and angiogenesis after ischemic stroke in rats.

## Methods

### Animals

Male Sprague–Dawley rats were purchased from Sino-British SIPPR/BK Laboratory Animal (Shanghai, China), and were housed at the Laboratory Animal Research Center of Zhejiang Chinese Medical University on a 12-h light/dark cycle with free access to water and food. All animal procedures were approved by the Experimental Animal Ethics Committee of Zhejiang Chinese Medical University (reference number: ZSLL-2014-37), Hangzhou, China and were conducted in accordance with the Guidelines for the Care and Use of Laboratory Animals established by the Science and Technology Committee of Zhejiang Province.

### Isolation, culture, and identification of BMSCs

BMSCs were harvested from 3-week-old Sprague–Dawley rats weighing 80–100 g. Briefly, the bone marrow was flushed from the femurs and tibias of rats with DMEM/F12 (Gino Bio-Pharm Technology, Hangzhou, China), 1% (v/v) penicillin and streptomycin (Haotian Biological Technology, Hangzhou, China). The cells were centrifuged and suspended in DMEM/F12 with 10% fetal bovine serum (FBS) (Gibco, Thermo Fisher Scientific, MD, USA), and cultured in 5% CO_2_ at 37 °C. After 48 h of incubation, the medium was changed and replaced every 3 days thereafter. When BMSCs reached 80–90% confluence, they were digested with 0.05% trypsin–EDTA (Gino Bio-Pharm Technology) and were subcultured at a ratio of 1:2. BMSCs from passage 3 were used in all experiments.

For identification of BMSCs, cell surface markers were analyzed by flow cytometry (Beckman-Coulter Inc., CA, USA). The cells were incubated with fluorescein isothiocyanate-conjugated antibodies against CD90, CD29, CD45 (Biolegend, San Diego, CA, USA), CD34 (Santa Cruz, CA, USA) and PBS (negative control) in a black chamber at 4 °C for 30 min. At least 5 × 10^5^ cells per sample were acquired and analyzed.

### Preconditioning of BMSCs with TMP in vitro

When BMSCs reached 70–80% confluence, the cells were pretreated with gradient concentrations of TMP (Aladdin, Shanghai, China) (0, 10, 25, 50, 100, and 200 μM) for 24 h under a 37 °C and 5% CO_2_ environment.

### Transwell migration assay

The migration assay was performed using transwell plates with pore size of 8 μm (Corning Costar, Cambridge, MA, USA). The upper chambers were loaded with 8 × 10^4^ BMSCs in 200 μl of DMEM/F12 containing 5% FBS. The lower chambers were loaded with 600 μl of DMEM/F12 containing 5% FBS, and 100 ng/ml SDF-1 (Peprotech Inc., Rocky Hill, NJ, USA). The chambers were incubated at 37 °C and 5% CO_2_ for 8 h. The upper surface of the membrane was then gently scraped to remove the nonmigrated cells and washed with PBS. The membrane was then fixed in 4% paraformaldehyde for 10 min followed by staining with 0.5% crystal violet for 20 min. The number of migrated cells was determined by averaging five random fields per well. The experiments were performed in quadruplicate for each group. For inhibitor studies, BMSCs were treated with or without TMP, and then were incubated for 8 h with 100 μg/ml AMD3100 (Sigma Aldrich, St Louis, MO, USA), a specific antagonist of CXCR4.

### Quantitative reverse transcription-polymerase chain reaction

Total RNA was extracted from brain tissue using TRIzol Reagent (Invitrogen, Carlsbad, CA, USA) according to the manufacturer’s instructions. cDNA was produced from the total RNA using PrimeScript RT Master Mix (TaKaRa, Tokyo, Japan). Quantitative PCR was conducted using the SYBR Premix Ex Taq Kit (TaKaRa) on an iQ5 multiplex real-time fluorescence quantitative PCR instrument (Bio-Rad, Hercules, CA, USA). Primers for CXCR4 consisted of 5′-ACT CAA TTC CAT GAG CAG AG-3′ (forward) and 5′-CTT TGC GTA AGT GTT AGC TG-3′ (reverse). Primers for GAPDH (an internal control) consisted of 5′-ACA GCA ACA GGG T GG TGG AC-3′ (forward) and 5′-TTT GAG GGT GCA GCG AAC TT-3′ (reverse). The reaction conditions were as following: preheated at 95 °C for 5 min, followed by 40 cycles of denaturation at 95 °C for 30 s, annealing at 55 °C for 30 s, and extension at 72 °C for 30 s. Each sample was tested in triplicate, and relative gene expression data were analyzed using the 2^–ΔΔCT^ method.

### Western blot analysis

The total protein concentration of cells or tissue was analyzed using the bicinchoninic acid assay (BCA; Beyotime, Beijing, China) according to the manufacturer’s instructions. Proteins were electrophoresed on a sodium dodecyl sulfate (SDS)–10% polyacrylamide gel and were transferred onto polyvinylidene difluoride (PVDF) membranes (Bio-Rad). Subsequently, membranes were incubated at 4 °C overnight with primary antibodies of rabbit anti-CXCR4 (Santa Cruz), anti-SDF-1 (Santa Cruz), or mouse anti-β-actin antibody (Santa Cruz), followed by incubation with goat anti-rabbit IgG (Thermo Pierce, USA) or goat anti-mouse IgG (Thermo Pierce) secondary antibody at room temperature for 2 h. The membrane was visualized using a chemiluminescence enhanced detection kit (Thermo Pierce), and was exposed to X-ray films. The expression of CXCR4 and SDF-1 was then normalized to β-actin.

### Middle cerebral artery occlusion in rats

The middle cerebral artery occlusion (MCAO) was performed using a modified method initially described by Longa et al. [26]. Briefly, rats were anesthetized with 10% chloral hydrate (350 mg/kg body weight) via intraperitoneal injection. The right carotid artery, external carotid artery (ECA), and internal carotid artery (ICA) were carefully isolated. The ECA was ligated and cut with a small mouth. A 4-0 nylon was inserted through the right ECA and was gently advanced into the ICA up to a point approximately 18 ± 2 mm from the bifurcation. After occlusion for 90 min, the nylon suture was withdrawn to permit reperfusion. Sham-operated rats underwent the same procedure without the nylon suture. A heating lamp was used to maintain the rectal temperature around 37 °C during the surgical procedure.

### BMSCs labeling and cell transplantation

Before transplantation, BMSCs were incubated with 10 μmol/L BrdU (Sigma Aldrich) for 48 h. BMSCs were then preconditioned with 100 μM TMP for 24 h and with 20 μM AMD3100 for 6 h [27]. Twenty-four hours after MCAO, approximately 1 × 10^6^ BMSCs in 1 ml PBS were injected into the rat via the tail vein. The rats were then randomly divided into five groups (*n* = 12 rats per group): sham-operated control group; MCAO rats with injection of PBS (MCAO group); MCAO rats with transplantation of nonpreconditioned BMSCs (BMSC group); MCAO rats with transplantation of TMP-preconditioned BMSCs (TMP group); and MCAO rats with transplantation of TMP/AMD3100-preconditioned BMSCs (AMD3100 group).

### Neurological function evaluation

The neurological deficits were evaluated 1, 7, and 14 days after ischemia by the investigators who were blinded to the experimental design. The modified neurological severity score (mNSS) includes motor, sensory, reflex, and balance tests [7]. The mNSS was used to evaluate the sensorimotor deficits by grading the score on a scale of 0–18 (normal score, 0; maximal deficit score, 18). The inability to perform a test or the lack of a tested reflex scores one point. Thus, a higher score means higher severity of injury. The adhesive removal test was used to assess somatosensory deficit as described previously [7]. Briefly, two small pieces of adhesive-backed paper dots (of equal size, 113.1 mm^2^) were used as bilateral tactile stimuli occupying the distal-radial area on the wrist of each forelimb. The time to remove each stimulus from the forelimbs was recorded in five trials per day for each forepaw. After 3 consecutive days of training, all of the rats were able to remove the dots within 10 s and then were subjected to MCAO. The mean time to remove the left dot was recorded. Sensorimotor function was also evaluated by the corner test which involves both stimulations of the vibrissae (sensory/neglect) and rearing (motor response) [28]. Briefly, two boards (30 cm × 20 cm × 1 cm) were set at a 30° angle with a small opening along the joint. A rat was placed between two boards facing the corner. When the rat entered deeply toward the corner, the two boards together stimulated both sides of the vibrissae. The rat then reared forward and upward, after which it turned back to face the open end. Ten trials were performed for each rat, and the selected sides for turning were noted.

### Immunofluorescence staining

The rats were sacrificed 14 days after MCAO by an overdose of 10% chloral hydrate. The rats were perfused transcardially with ice-cold saline, and then fixed by perfusion with 4% paraformaldehyde solution. The brains were removed and kept in 4% paraformaldehyde solution overnight at 4 °C, and then soaked in 30% sucrose solution. The brain tissue was cut into 10-μm-thick frozen sections (Leica CM1950; Leica Microsystems, Wetzlar, Germany). BrdU-labeled BMSCs were detected by immunofluorescence staining. Briefly, the sections were first pretreated to denature DNA as follows: after immersion in 50% formamide/2× saline-sodium citrate buffer (SSC) at 65 °C for 2 h, the sections were washed in PBS for 10 min, incubated in 2 N HCl at 37 °C for 30 min, and washed with 0.1 M boric acid (pH 8.5) for 10 min. The sections were then incubated with a mouse anti-BrdU (1:100; Sigma Aldrich) at 4 °C overnight, washed in PBS, and then incubated with secondary antibodies of Alexa Fluor 555-conjugated goat anti-mouse IgG (Thermo Fisher Scientific Inc., MA, USA) for 1 h at 37 °C. Counterstaining was done with 4′,6-diamidino-2-phenylindole (DAPI; Zhongshan Golden Bridge Biotechnology, Beijing, China). For angiogenesis analysis, the tissue sections were washed twice in PBS and incubated in 0.3% Triton X-100 for 30 min, incubated in 3% H_2_O_2_ for 20 min and then in 5% goat serum for 1 h, and stained with rabbit anti-von Willebrand factor (vWF; Santa Cruz). Fluorescein isothiocyanate (FITC)-conjugated goat anti-rabbit IgG (Santa Cruz) was used as the secondary antibody.

### Statistical analysis

Data were presented as mean ± standard deviation (SD) and were analyzed using SPSS software (version 17.0; SPSS Inc., Chicago, IL, USA). mNSS was analyzed by nonparametric Mann–Whitney *U* test. One-way analysis of variance followed by the Tukey’s post-hoc test was used for analyzing parametric data and multiple comparison, and *P* <0.05 was considered statistically significant.

## Results

### Identification of BMSCs

**Fig. 1:**
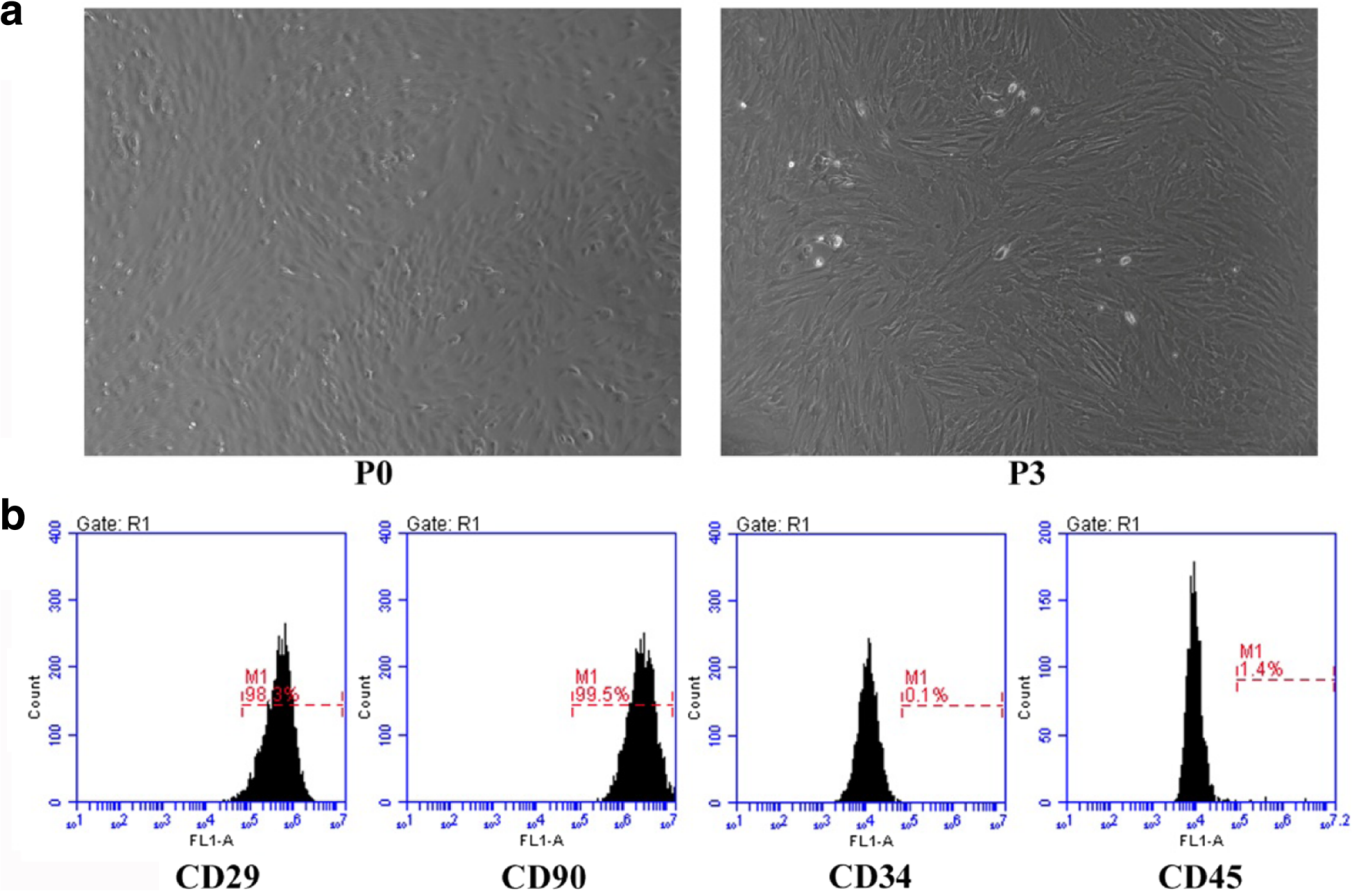
Identification of BMSCs. **a** P0 BMSCs showed short bar shapes, and P3 BMSCs adopted a uniformly spindle-shaped population. **b** Flow cytometry revealed that BMSCs expressed the surface markers CD29 and CD90, but not CD34 and CD45. *﻿BMSCs* ﻿bone marrow-derived mesenchymal stem cells﻿,﻿ *P0* passage 0, *P3* passage 3

The primary BMSCs cultured in the culture dish displayed short bar shapes initially. Passage 3 (P3) BMSCs showed typical spindle-shaped cell morphology (Fig. 1a). Flow cytometry analysis confirmed that these cells were positive for mesenchymal cell markers CD29 (98.3%) and CD90 (99.5%), but negative for hematopoietic cell markers CD34 (0.1%) and CD45 (1.4%) (Fig. 1b), suggesting that the cultured cells had similar morphological and immunophenotypical characteristics of BMSCs.

### Preconditioning with TMP enhances BMSCs migration in vitro

**Fig. 2:**
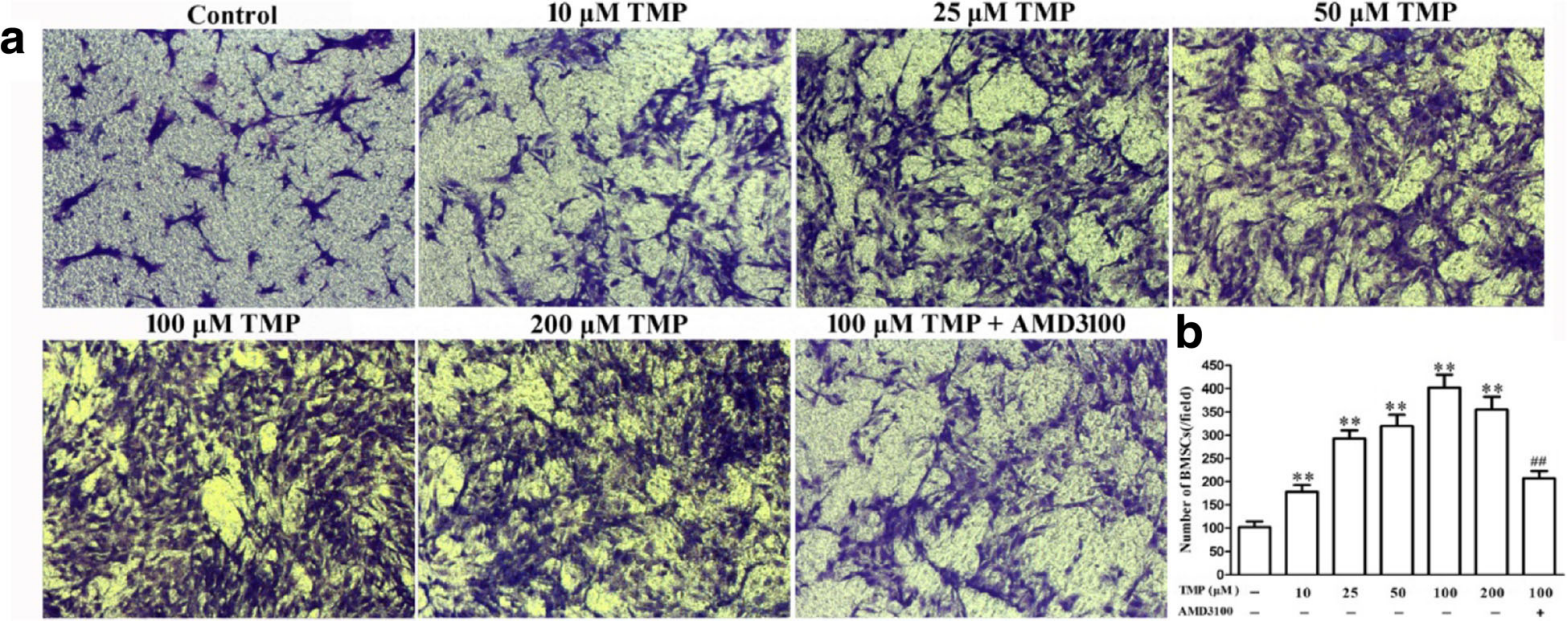
TMP enhanced BMSCs migration in vitro. **a** Representative images of migrated BMSCs in the transwell assay (magnification × 200). **b** Quantification of transwell results. Values are mean ± SD. **P < 0.01 vs. control. ^##^ *P* < 0.01 vs. 100 μM TMP. *BMSCs* bone marrow-derived mesenchymal stem cells, *TMP* tetramethylpyrazine

A transwell system was used to investigate the effect of TMP preconditioning on BMSCs migration in vitro. Using SDF-1 as a chemotactic agent in the lower chamber, preconditioning with TMP (0, 10, 25, 50, 100, and 200 μM) for 24 h concentration-dependently increased the BMSCs migratory capacity, especially in the 100 μM TMP group (Fig. 2). Most importantly, cotreatment of BMSCs with AMD3100 (100 μg/ml), a CXCR4 antagonist, significantly inhibited the effects of TMP on cell mobility (Fig. 2). These results indicated that preconditioning with TMP promoted BMSCs migration through SDF-1/CXCR4 pathway.

### Preconditioning with TMP enhances CXCR4 expression in BMSCs

**Fig. 3:**
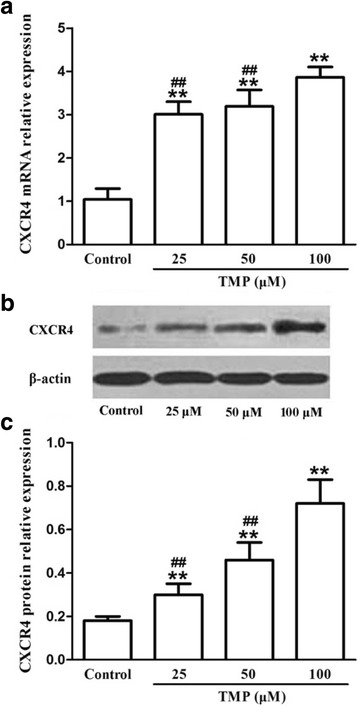
TMP enhanced CXCR4 mRNA and protein expression in BMSCs in vitro. **a** TMP dose-dependently increased CXCR4 mRNA expression by qRT-PCR. **b** Representative western blot analysis showed that TMP enhanced CXCR4 protein expression in BMSCs. **c** Semiquantitative analysis of CXCR4 protein. Values are mean ± SD. **P < 0.01 vs. control. ^##^ *P* < 0.01 vs. 100 μM TMP. *TMP* tetramethylpyrazine, *BMSCs* bone marrow-derived mesenchymal stem cells, *qRT-PCR* quantitative reverse transcription-polymerase chain reaction, *CXCR4* CXC chemokine receptor 4

To test whether TMP preconditioning could enhance BMSCs migration via upregulating CXCR4 expression, the CXCR4 expression was detected by quantitative reverse transcription-polymerase chain reaction (qRT-PCR) and western blot analysis. The results showed that TMP preconditioning for 24 h significantly increased CXCR4 mRNA (Fig. 3a) and protein (Fig. 3b, c) expression in BMSCs in a concentration-dependent manner. These data demonstrated that TMP enhanced BMSCs migration by upregulating the expression of CXCR4.

### Transplantation of BMSCs preconditioned with TMP improves neurological function recovery

**Fig. 4:**
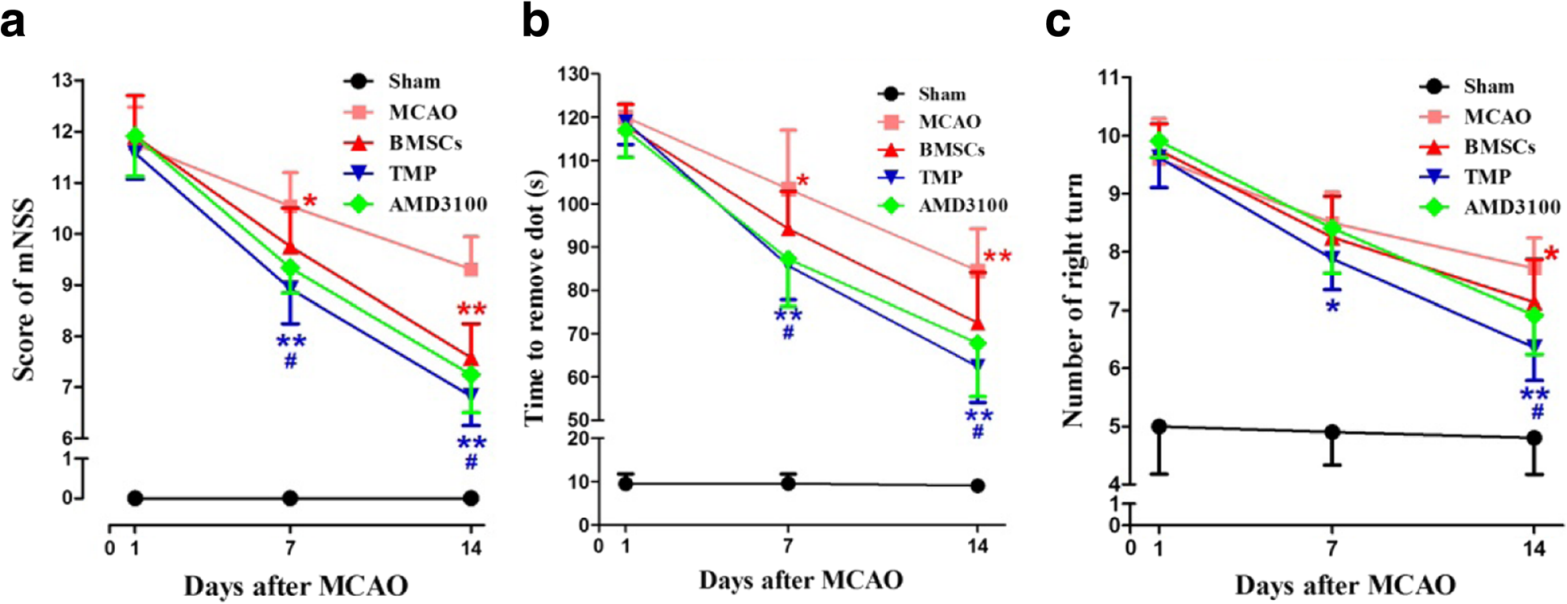
BMSCs preconditioned with TMP improved functional recovery in MCAO rats. **a** mNSS test. **b** Adhesive removal test. **c** Corner test. ^*^ *P* < 0.05, **P < 0.01 vs. MCAO. #*P* < 0.05 vs. AMD3100. *BMSCs* bone marrow-derived mesenchymal stem cells, *MCAO* middle cerebral artery occlusion, *mNSS* modified neurological severity scores, *TMP* tetramethylpyrazine

To determine whether BMSCs preconditioned with TMP (100 μM) improved neurological function recovery, the mNSS, the adhesive removal test, and the corner test were performed 1, 7, and 14 days after MCAO. BMSCs preconditioned with TMP significantly improved the functional outcome compared with the BMSC group at 7 and 14 days (Fig. 4), respectively. However, these beneficial effects of TMP preconditioning were reduced by AMD3100 (Fig. 4a–c).

### Preconditioning with TMP enhances BMSCs homing into the ischemic brain

**Fig. 5:**
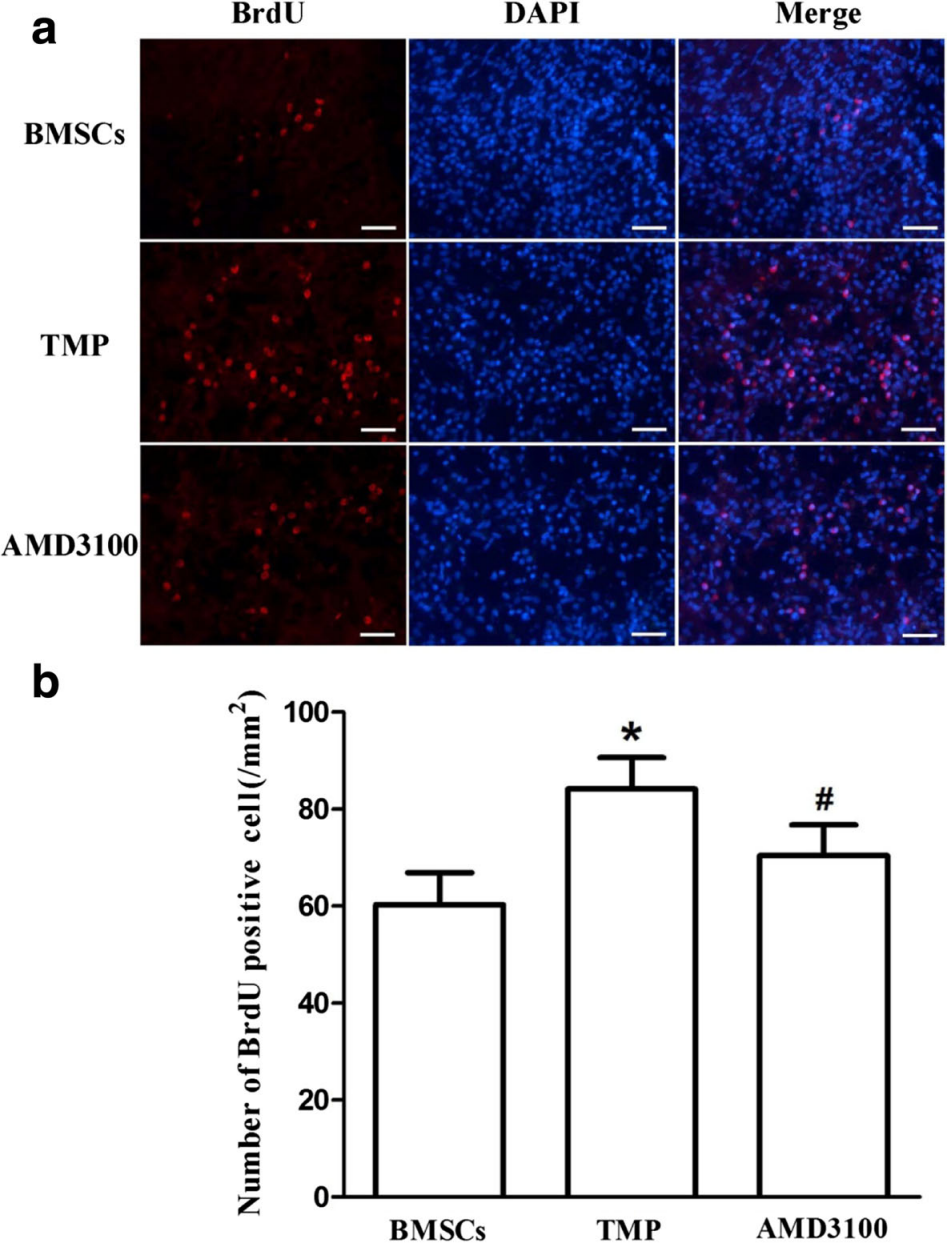
BMSCs preconditioned with TMP enhances BMSCs homing into the ischemic brain region. **a** BMSCs that were labeled by BrdU (*red*) and nuclei labeled with DAPI (*blue*) were observed at the lesion site after MCAO. **b** Quantification of migrated BMSCs. *Bars*, 100 μm. Values are mean ± SD. *P < 0.01 vs. BMSCs. #*P* < 0.01 vs. TMP. *BMSCs* bone marrow-derived mesenchymal stem cells, *TMP* tetramethylpyrazine, *MC﻿AO* middle cerebral artery occlusion, *BrdU* 5-bromo-2-deoxyuridine, *DAPI* 4′,6-diamidino-2-phenylindole (Color figure online)

To detect the homing efficiency of injected BMSCs, the number of BrdU-positive BMSCs was counted in the ischemic regions. In the penumbra cortex, TMP preconditioning increased BMSCs homing efficiency compared with the BMSCs group on day 14 after MCAO (Fig. 5a, b). However, copreconditioning with the CXCR4 antagonist AMD3100 largely inhibited the effects of TMP (Fig. 5a, b).

### Transplantation of BMSCs preconditioned with TMP increases angiogenesis in ischemic regions

**Fig. 6:**
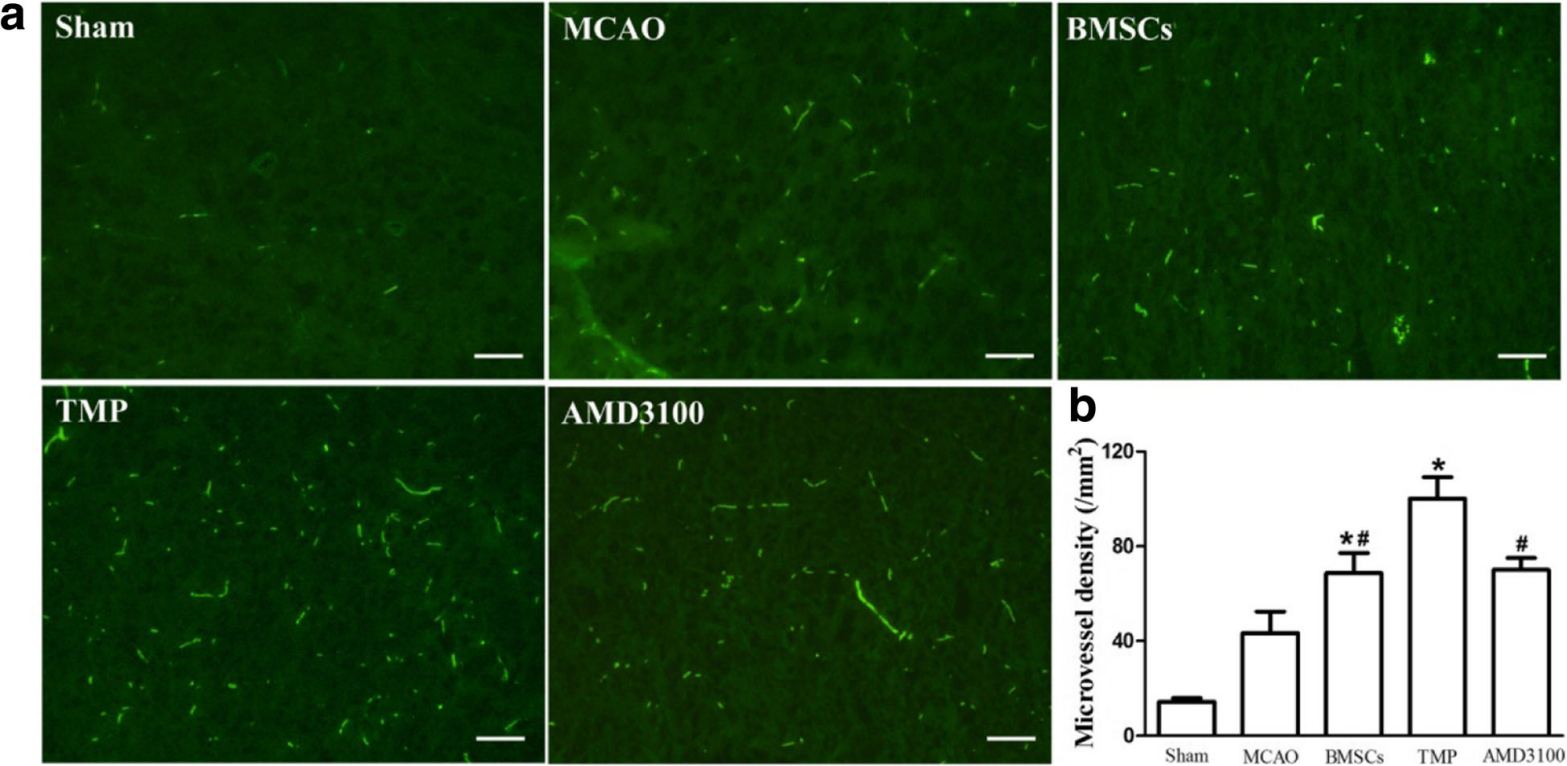
BMSCs preconditioned with TMP increased microvessel density in ischemic brain regions. **a** Microvessel density determined by vWF immunofluorescence staining. **b** Quantification of microvessel density. *Bars*, 50 μm. Values are mean ± SD. *P < 0.01 vs. MCAO. #*P* < 0.01 vs. TMP. *BMSC* bone marrow-derived mesenchymal stem cell, *vWF *Von Willebrand factor, *TMP* tetramethylpyrazine, *MCAO* middle cerebral artery occlusion

Microvessel density was analyzed in the penumbra cortex on day 14 after MCAO. Compared with the MCAO group, BMSCs transplantation significantly increased microvessel density. Moreover, MCAO rats transplanted with BMSCs preconditioned with TMP had the highest microvessel density (Fig. 6a, b). However, copreconditioning with AMD3100 partially eliminated the effects of TMP (Fig. 6a, b). These data indicated that TMP-preconditioned BMSCs increased angiogenesis in the ischemic regions.

### Transplantation of BMSCs preconditioned with TMP promotes protein expression of SDF-1 and CXCR4 in ischemic regions

**Fig. 7:**
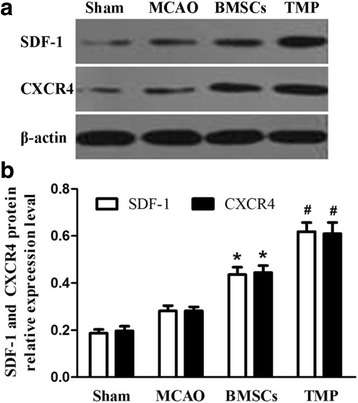
BMSCs preconditioned with TMP increased SDF-1 and CXCR4 expression in ischemic brain regions. **a** Expression of SDF-1 and CXCR4 was determined by western blot analysis. **b** Semiquantitative analysis of the SDF-1 and CXCR4 protein. Values are mean ± SD. *P < 0.01 vs. MCAO, #*P* < 0.01 vs. BMSCs. *BMSCs* bone marrow-derived mesenchymal stem cells, *TMP* tetramethylpyrazine, *SDF-1* Stromal cell-derived factor-1, *CXCR4* CXC chemokine receptor-4, *MCAO* middle cerebral artery occlusion

The protein levels of SDF-1 and CXCR4 were detected by western blot analysis. Expression of SDF-1 and CXCR4 was significantly increased in the BMSC and TMP-preconditioned groups compared with the MCAO group (Fig. 7a, b). Compared with the BMSC group, the protein expression levels of SDF-1 and CXCR4 were significantly increased in the TMP-preconditioned group (Fig. 7a, b). These data indicated that TMP preconditioning stimulated the SDF-1/CXCR4 axis and enhanced BMSCs migration to the ischemic regions.

## Discussion

In the present study, we found that TMP enhanced BMSCs migration and increased CXCR4 mRNA and protein expression in vitro. We then demonstrated that TMP preconditioning before transplantation improved BMSCs homing toward ischemic brain after MCAO treatment, which was accompanied by improved behavioral performance and angiogenesis in the penumbra cortex. However, these effects were largely abolished by the CXCR4 antagonist AMD3100, indicating that the benefits of TMP preconditioning are mediated by the SDF-1/CXCR4 axis. To the best of our knowledge, the current study is the first to investigate the pro-migratory effect of TMP on BMSCs in vitro and in an experimental ischemic model of stroke.

MSCs-based therapy has been shown to improve neurological outcome after ischemic stroke in rodent models and clinical trials [3, 5–7]. It is commonly recognized that a successful cell-based therapy for stroke depends largely on cell homing and engraftment to the brain ischemic region, especially when cells are infused via the vascular route [29]. However, the low migration ability of transplanted MSCs toward the ischemic region limits the efficacy of this approach [8, 9]. Therefore, the development of strategies that enhance the migration of MSCs is the key to optimize MSC therapy in stroke. Recently, transplantation of MSCs preconditioned with different treatment, such as hypoxia, cytokines, chemical drugs, and genetic modifications, has shown much better engraftment and efficacy [10]. Preconditioning with the mood stabilizers valproate (VPA) and/or lithium (Li) promoted the migration and homing ability of MSCs and improved functional recovery in a stroke model [27, 30]. In the present study, we found that TMP preconditioning enhanced the migration and homing ability of BMSCs and improved neurological function recovery.

The SDF-1/CXCR4 axis has been reported to contribute to the migration of MSCs into damaged tissues [13–15]. SDF-1 expression was higher in the ischemic boundary zone after stroke, which may facilitate the migration of CXCR4-positive stem cells [14, 16]. CXCR4, the specific receptor of SDF-1, is highly expressed in BMSCs in the bone marrow. However, its expression is markedly reduced in culture-expanded BMSCs, leading to decreased migration toward the SDF-1 gradient [19, 31]. Thus, approaches to increase the expression of CXCR4 can enhance the migration ability of BMSCs. Genetic approach is an effective method to increase the expression of CXCR4 in MSCs. CXCR4 overexpression with lentiviral transduction in the MSCs promoted their migration and enhanced neuroprotection in a rat model of cerebral ischemia [32]. However, genetic modification seems infeasible in current clinical practice. Unlike genetic engineering approaches, pharmacological preconditioning is a simple and clinically feasible approach. Tsai et al. [27, 30] demonstrated that preconditioning with the mood stabilizer valproic acid (VPA) promoted the homing and migration ability of MSCs through upregulation of CXCR4 expression. Similarly, treatment with tanshinone IIA and astragaloside IV enhanced the ability of MSCs to home to ischemic myocardium sites by increasing the expression of CXCR4 [33]. TMP is one of the most important active components of a traditional Chinese herb from *Ligustium wallichii* Franchat, which has been widely used in China for the treatment of ischemic stroke [20–22]. Consistent with previous studies [23–25], we found that increased CXCR4 expression with TMP pretreatment enhanced the migration of BMSCs toward SDF-1 in an in vitro transwell system, and promoted the homing of BMSCs toward ischemic brain following intravenous administration in vivo. These beneficial effects of TMP were largely reversed by the CXCR4 antagonist AMD3100 in vitro and in vivo, further confirming that the SDF-1/CXCR4 axis regulates BMSC migratory behavior.

The mechanism of BMSC therapy for ischemic stroke primarily includes cell replacement, neuroprotection, angiogenesis, and neurogenesis [7, 34, 35]. The functional benefits after BMSC transplantation have been attributed to increased angiogenesis after ischemic stroke [35, 36]. BMSCs promote angiogenesis through release of angiogenic factors and chemokines, such as vascular endothelial growth factor (VEGF), angiopoietin-1 (Ang1), basic fibroblast growth factor (bFGF), and SDF-1 [35–37]. SDF-1 not only facilitates the homing of transplanted BMSCs, but also increases vascular density and induces neovascularization in the ischemic tissue [37–39]. In the present study, we demonstrated that TMP preconditioning enhanced BMSC engraftation, upregulated SDF-1 expression, and promoted angiogenesis in the ischemic brain. In addition, the number of engrafted BMSCs was closely associated with angiogenesis after stroke. Our findings suggest that TMP preconditioning may enhance the mobilization of BMSCs into the ischemic brain, and engraftment of TMP-preconditioned BMSCs promotes angiogenesis in the ischemic boundary zone. These changes significantly promoted the recovery of neurological function in rats with cerebral ischemia, which may have important clinical implications.

## Conclusions

TMP preconditioning enhances the migration and homing ability of BMSCs and improves neurological performance after stroke. The combination of SDF-1 with CXCR4 may contribute to the trafficking of transplanted BMSCs. Our results suggest that TMP preconditioning enhances the neuroregenerative effects of BMSCs and could be a beneficial strategy to improve the therapeutic potential of cell transplantation for ischemic stroke in the clinical setting.

## Abbreviations

BMSCs: Bone marrow-derived mesenchymal stem cells
BrdU: 5-Bromo-2-deoxyuridine
BSA: Bovine serum albumin
CXCR4: CXC chemokine receptor 4
DMEM: Dulbecco's modified Eagle's medium
ECA: External carotid artery
FBS: Fetal bovine serum
GAPDH: Glyceraldehyde-3-phosphate dehydrogenase
ICA: Internal carotid artery
MCAO: Middle cerebral artery occlusion
mNSS: Modified neurological severity score
MSCs: Mesenchymal stem cells
PBS: Phosphate-buffered saline
qRT-PCR: Quantitative reverse transcription-polymerase chain reaction
SD: Standard deviation
SDF-1: Stromal cell-derived factor-1
TMP: Tetramethylpyrazine
vWF: Von Willebrand factor
